
JOURNAL INFORMATION
==============================
NLM Title Abbreviation: ScientificWorldJournal
Journal ID: ScientificWorldJournal
NLM Journal ID: 101131163
Journal ID: TSWJ

The Scientific World Journal

ISSN: 2356-6140
EISSN: 1537-744X
Publisher: Wiley

ARTICLE INFORMATION
==============================
PMCID: PMC6084253
PMID: 30147518
DOI: 10.1100/tsw.2001.164
Article ID: 721837
Article version: 1
Subjects: Short Report

Real Time Monitoring of Apoptosis, Induced by Beta-Amyloid Peptide, in SH-SY5Y Neuroblastoma Cells Expressing a GFP-Tagged Nuclear Pore Protein

Holmlund Linda
Imreh Gabriela
Hallberg Einar
Iverfeldt Kerstin *
Department of Neurochemistry and Neurotoxicology, Stockholm University, S-106 91, Stockholm, and Södertörns University College, S-141 04 Huddinge, Sweden
*Kerstin Iverfeldt: kerstin@neurochem.su.se
Publication date: 2001
Electronic publication date: 2001 Oct 30
Volume: 1
First page: 54
Last page: 54
Copyright: Copyright © 2001 Linda Holmlund et al.
Copyright year: 2001
License: This is an open access article distributed under the Creative Commons Attribution License, which permits unrestricted use, distribution, and reproduction in any medium, provided the original work is properly cited.
License URL: https://creativecommons.org/licenses/by/3.0/

Funding: Swedish National Board for Laboratory Animals

==============================
Miami *Nature Biotechnology* Short Reports
*TheScientificWorld* (2001) 1(S3), 54SR
ISSN 1532-2246; DOI 10.1100/tsw.2001.164

### REAL TIME MONITORING OF APOPTOSIS, INDUCED BY BETA-AMYLOID PEPTIDE, IN SH-SY5Y NEUROBLASTOMA CELLS EXPRESSING A GFP-TAGGED NUCLEAR PORE PROTEIN

Linda Holmlund, Gabriela Imreh, Einar Hallberg, and Kerstin Iverfeldt*

Department of Neurochemistry and Neurotoxicology, Stockholm University, S-106 91, Stockholm, and Södertörns University College, S-141 04 Huddinge, Sweden
* kerstin@neurochem.su.se

**INTRODUCTION.** Previously it was reported that during apoptosis the GFP (green fluorescent protein) fluorescence disappeared from a neuroblastoma cell line overexpressing the integral nuclear pore membrane protein POM121 tagged with GFP (1). The Alzheimer-associated peptide beta-amyloid, which forms senile plaques in the brain of Alzheimer patients, has been shown to have neurotoxic properties and to induce apoptosis. In this study we have used human SH-SY5Y neuroblastoma cells transfected with POM121-GFP to investigate the proapoptotic properties of beta-amyloid peptides.

**METHOD.** Transfected SH-SY5Y cells (1), grown on cover slips, were treated with 1 µM retinoic acid (RA) in serum-free medium for 3 days. The cover slips were mounted on a chamber containing beta-amyloid peptide in serum-free medium and the time-lapse experiments were immediately started. GFP fluorescence was analyzed by fluorescence microscopy in parallel with phase contrast microscopy analysis of the morphological appearance.

**RESULTS.** The transfected neuroblastoma cells were differentiated into post mitotic cells in the presence of RA, which also induced neurite outgrowth. Morphological effects on the cells were evident already within one hour after exposure to the beta-amyloid (25-35) peptide (25 µM) and the fluorescence was almost completely abolished within 4 hours (see Fig. 1). The results indicate that the transfected neuroblastoma cells are as sensitive as primary cultures of neuronal cells to induction of apoptosis by beta-amyloid peptides, and that monitoring the disappearance of the nuclear rim fluorescence is a sensitive method to detect apoptosis within hours after exposure.

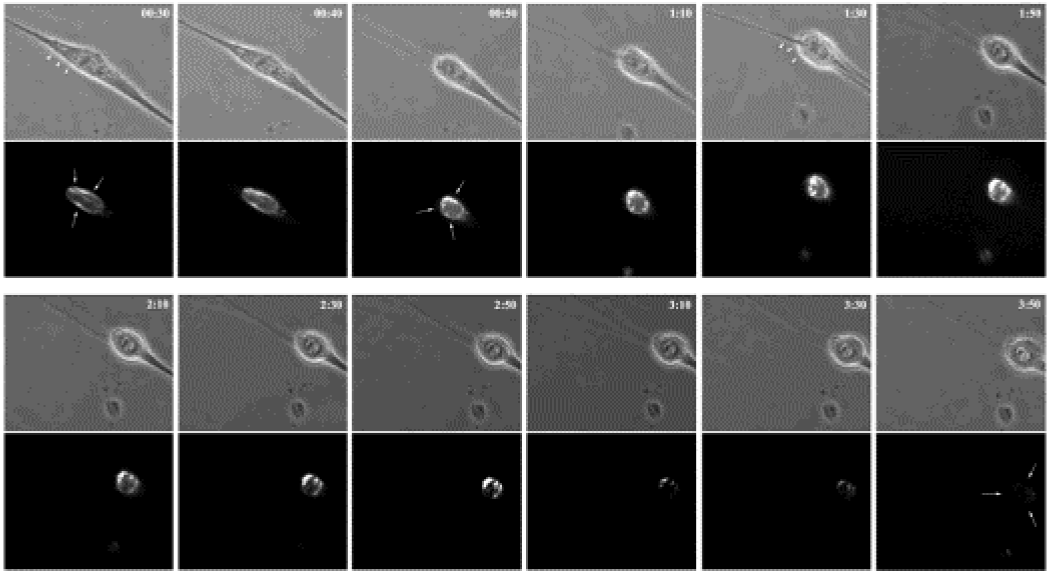

**Fig. 1.** Time-lapse study of beta-amyloid induced apoptosis in a human SH-SY5Y neuroblastoma cell line expressing POM121-GFP. The cells were incubated at 37° C in cell culture medium containing 25 µM beta-amyloid (25-35). Every 10 min a phase contrast (upper panels) and a fluorescence (lower panels) micrograph were taken. The time after addition of the peptide is indicated in the upper right corner of each phase contrast micrograph. Note the change in morphology (arrowheads) concomitant with the disappearance of the GFP fluorescence (arrows) in the periphery of the nucleus.

**ACKNOWLEDGEMENT.** This work was supported by grants from the Swedish Natural Board for Laboratory Animals.

**REFERENCE.**

1. Imreh, G., Beckman, M., Iverfeldt, K., and Hallberg, E. (1998) Exp. Cell Res. 238, 371-376
